# Book Reviews

**Published:** 1895-06

**Authors:** 


### Book Reviews.

CLINICAL GYNECOLOGY, MEDICAL AND SURGICAL, for Students and Practitioners. By Eminent American Teachers. Edited by JOHN M. KEATING, M. D., LL. D., and by HENRY C. COE, M. D., M. R. C. S., Professor of Gynecology, New York Polyclinic. Illustrated. Octavo, pp. xviii.—994. Philadelphia: J. B. Lippincott Co. 1895.

Without attempting to discuss the propriety of the appearance at this time of another treatise on gynecology, we shall proceed to an examination of this work on its merits.

The book opens with an introductory chapter by the late Dr. William Goodell. While this is interesting in many respects, especially to those who have not read the author's "lessons," yet we are somewhat at a loss to reach an estimate of either its value, or the propriety of increasing the bulk of the book twenty-five pages for the purpose of affording a display of classic knowledge or facile writing.

In the first chapter of the treatise proper are considered the methods of gynecological examination and general outlines of differential diagnosis, by Drs. William H. Baker and Francis H. Davenport. These authors are quite competent to deal clearly, comprehensively and succinctly with these important subjects that present themselves at the very threshold of gynecological knowledge. That they have done so in the present instance may readily be determined by a careful examination of the important chapter that they have prepared. While many of the methods are those that are in constant use by intelligent gynecologists, particular interest centers around the examination of the ureters and bladder. Here the latest information on the subject will be found.

An elaborate section on gynecological technique is presented by Dr. Hunter Robb that is very completely illustrated. The impetus that bacteriological research has given to clean surgery is here unfolded in all its amplifications, yet we think it is carrying the matter to an extreme for an author to recommend an especially constructed razor in an elaborate case to which he attaches his name. The fact is that there is too great a multiplication of instruments and paraphernalia connected with gynecological and surgical practice. There should be on all hands a greater attempt to simplify. Few instruments, scrupulous cleanliness, minute attention to detail and careful preparation are the cardinal points of success that should be carefully pointed out and insisted upon by surgical and gynecological teachers.

The third chapter gives an outline of gynecological therapeutics and is written by Dr. Bache McE. Emmet. Here is an opportunity for much sound suggestion and teaching, and Dr. Emmet proves himself quite competent to deal with the subject that he has undertaken on the lines indicated. It is important that young girls, as they approach puberty, should be subjected to more hygienic care than has been usual ; and especially is it important that their school life should receive discreet supervision. Here is afforded the greatest field that preventive medicine, as applied to gynecology, possesses. We could wish that Dr. Emmet had enlarged upon this portion of the subject, but, in the main, he has done well—better than most authors in similar treatises.

In the next chapter we find considered anomalies of development in the genital tract, by Dr. Barton Cook Hirst, a section which is interesting alike to the biologist and teacher. While it is necessary, to complete such a treatise as this, to introduce this history, and is a curiously interesting part of it, it has less practical importance than most of the other sections.

In the fifth chapter are considered traumatic lesions of the vulva, vagina and cervix, by Dr. Matthew D. Mann, of Buffalo. While this is a well-written chapter, its especial strength, to us, seems to be in its operative technique. It is very brief in its consideration of the injuries due to external violence and coitus, though more elaborate in those caused by parturition. It seems to us that this chapter might have been much extended with interest and profit by so experienced a teacher as its distinguished author.

A chapter that will attract considerable attention is that on inflammation of the female genital organs, written by Dr. William M. Polk, of New York. This is especially the case of that portion of it which deals with pyosalpinx and its correlated conditions. Collections of pus within the pelvic cavity has especial interest nowadays, in view of the recent revelations concerning gonorrhea as a causative factor of pelvic inflammation. It is a matter of some doubt whether there are any pelvic pus collections that are not due either to gonorrhea, parturition, abortion, ectopic pregnancy, or unclean hands or instruments. There is not much use in wasting time over the old-fashioned pelvic cellulitis. When pus exists in the pelvis it generally must be sought by abdominal section ; exceptionally through the vagina. The latter has been termed most aptly as the Dismal Swamp method.

Genital tuberculosis is considered in the seventh chapter by Dr. J. Whitridge Williams, of Baltimore. Recently this subject has attracted attention among surgeons and gynecologists, hence a chapter on this subject by so competent an author finds an appropriate place in this volume.

In the next chapter we find inflammatory lesions of the pelvic peritoneum connective tissue well handled by Dr. Henry T. Byford, of Chicago. It is astonishing how easy it is to write out a good diagnosis of these lesions in a text-book, but it is amazingly difficult to differentiate them at the bedside. We hope this chapter may aid in solving this difficult problem for some of its readers.

The next subject is displacements of the uterus, which is considered by Dr. Paul F. Mundé, of New York. This author is especially fitted to handle this subject well, and he has drawn upon a large experience in setting it forth. The illustrations are well adapted to make the text clear, and altogether it is a most satisfactory exposition of the subject.

In the following three chapters, X., XI. and XII., are considered respectively, neoplasms of the vulva and vagina, benign neoplasms of the uterus, and malignant neoplasms of the uterus, all written by Dr. Hermann J. Boldt, of New York. These subjects form one of the most important, as well as the most interesting, departments in gynecological literature, and this author has done himself great credit in the admirable setting forth that he has given them.

Not second to the foregoing subject is that of neoplasms of the ovaries, tubes and broad ligaments, which is ably considered in Chapter XIII. by the surviving editor, Dr. Henry C. Coe, of New York. This and the three preceding chapters furnish the subject for an entire treatise, and it is along these lines that we should approve of a new treatise in the gynecological field. Already material enough has accumulated, and we could wish it would be put in permanent form in a separate treatise for study and reference. Dermoid cysts furnish a most interesting study, and we think Dr. Coe might have elaborated this subject with considerable profit. Complications of ovarian tumors is also an interesting study, especially when associated with pregnancy. The propriety of operating in these latter cases is no longer doubted.

The chapter on ectopic pregnancy is written by Dr. William T. Lusk, of New York, who is an experienced author and teacher, but we think that he has hardly met expectation in this instance. This section could have been considerably elaborated in pathology as well as operative treatment, hence it follows that an operator of large experience might better have written it.

Passing on, we note that functional diseases are considered by Dr. Chauncey D. Palmer, of Cincinnati, in a well-prepared chapter on that subject. Diseases of the urethra, bladder and ureters are considered in Chapter XVI., by Drs. Charles Jewett and John O. Polak. In this we find the old familiar pictures that have done duty in the journals and *Twentieth Century Practice* prepared by the gynecologist of Johns Hopkins Medical School. Diseases of the rectum and anus are considered by Dr. E. E. Montgomery and many of the illustrations in this chapter have been published in the transactions of the American Association of Obstetricians and Gynecologists and in the JOURNAL.

One of the most important chapters in the book, whether estimated from its surgical value or scholarly treatment, is that on diseases of the female breast, by Dr. Dudley P. Allen, of Cleveland. Every surgeon will appreciate this section, which is a valuable addition to the literature of the subject.

The volume closes with a chapter on cutaneous diseases peculiar to women, written by Drs. Lewis A. Duhring and Milton B. Hartzell.

During the preparation of this work, the senior editor, Dr. Keating, died, which devolved the delicate task of its completion upon Dr. Coe. That he has performed his part ably and satisfactorily it is pleasant to state. His part has been a difficult one, and it is not easy to discover how he could have improved upon his opportunity. This volume is handsomely illustrated in many respects, and must be regarded as a distinct addition to gynecological literature, but its chief claim to the favor of the profession will be through the fact that it is a symposium of the American Gynecological Society.

---

Surgical Pathology and Therapeutics. By John Collins Warren, M. D., Professor of Surgery, Medical Department Harvard University; Surgeon to the Massachusetts General Hospital, etc. Octavo volume, pp. 832, with 135 illustrations. Price, cloth, $6; half-morocco, $7.00 net. Philadelphia: W. B. Saunders, 925 Walnut street. 1895.

It has been necessary to reconstruct the most important or foundation principles of surgical pathology since the germ theory of disease has become established through the science of bacteriology. Hence it is not at all remarkable that we find the first seventy-eight pages of this work exclusively devoted to the consideration of the study of microörganisms. But the subject does not end here; it pervades almost every chapter in the book, and especially is it well brought out in the sections on infective inflammation. The author's treatment of this latter topic may be regarded as a surgical classic. It affords the most delightful reading on the subject that has ever fallen to our vision, and almost every sentence is instructive.

Tuberculosis is taken up in Chapter XXIV., and here again the author stands out in bold relief by his original treatment of the subject, to the consideration of which, in its multifarious forms, he devotes nearly 100 pages. When we reflect upon its importance, this is not too much space to allot to its discussion. Only lately has tuberculosis begun to attract surgeons, but already it has become of absorbing interest to them. Warren asserts that the surgeon has, in fact, more to do with the disease today than the physician, which is a surgical truism. He tells us that the inoculability of tuberculosis was first recognized by Laënnec in 1826, who, himself, fell a victim to its surgical infection. Villemin, in 1865, first demonstrated experimentally the possibility of its transmission from man to animals, while Koch's discovery, in 1882, fairly revolutionized this great department of surgery. Warren's observations on the treatment of tuberculosis of bones and joints is the best surgical exposition of the subject we have seen, while his chapter on tuberculosis of the soft parts, in many respects, is unique. The chapter on aseptic and antiseptic surgery, which is placed at the end of the book, should be read by every physician as well as surgeon. It is a fitting anti-climax to the opening chapters on bacteriology. In an appendix Warren briefly considers several surgical topics of interest.

To appreciate this book it should be read, for then its pure English and delightful rhetoric will charm every lover of good medical literature, while it teems with sufficient originality, either of thought or practice, to become a necessity for every surgeon. It is handsomely printed, and some of its illustrations, many of which are quite new, are especially fine.

---

MATERIA MEDICA AND THERAPEUTICS FOR PHYSICIANS AND STUDENTS. By JOHN B. BIDDLE, M. D., late Professor of Materia Medica and General Therapeutics in the Jefferson Medical College, Philadelphia. Thirteenth edition, revised, re-arranged and enlarged, with special reference to therapeutics, toxicology, the physiological action of medicines and containing all the preparations and remedies described in the United States Pharmacopeia of 1890, to which the work has been made to conform. By Clement Biddle, M. D., Medical Corps, United States Navy. With numerous illustrations. Octavo, pp. 714. Price, $4.00. Philadelphia: P. Blakiston, Son & Co., 1012 Walnut street. 1895.

This treatise has been before the profession so long that it may be regarded as an old friend. It was first issued in 1865; hence, for thirty years it has been used as a text-book or a work of reference. The author died in 1879 and subsequent editions have been edited by Dr. Henry Morris and Dr. Clement Biddle of the United States Navy. The ninth, tenth and eleventh editions were written conjointly by these two; the twelfth and thirteenth by the latter alone.

In the present edition the same order has been observed, whereever it has been practicable, as in the preceding one so far as the consideration of each drug or remedy is concerned. In presenting the physiological effects of drugs, the following plan has been followed—namely, first, local action comprising analgesic, irritant, antiseptic and pupillary effects; second, internal action beginning with taste and following with effects upon the stomach, secretions, nervous, vascular, respiratory and muscular systems, temperature and elimination.

This edition is intended to include all preparations considered in the United States Pharmacopeia of 1890, hence the newer additions to the list will be found in this book. Various changes have also found place, especially as regards the application of remedies to the eye and throat, the dispensing of disagreeable medicines in capsules and tablet triturates, the kinds of enemata in use and instructions for making poultices. A new order termed protectants and absorbents is introduced under the head of topical medicines, and the ferruginous preparations are transferred from tonics to hematinics.

This edition is increased seventy-five pages, by the addition of fifteen pages to the work proper and sixty pages to the appendix.

An amplified index to contents and to diseases and remedies has received careful revision, increasing its value and the ease with which it can be referred to. The editor especially desires that one discovering errors in the book will report the same to him, that corrections may be made in future editions. Any such will be duly acknowledged.

The reputation of this work as one of the very best treatises on materia medica and therapeutics, which it has so long enjoyed, is fully sustained in this thirteenth edition.

---

Sexual Neurasthenia: Its Hygiene, Causes, Symptoms and Treatment, with a chapter on Diet for the Nervous. By George M. Beard, A. M., M. D., formerly Lecturer on Nervous Diseases in the University of the City of New York; Fellow of the New York Academy of Medicine, etc., etc. Edited, with notes and additions, by A. D. Rockwell, A. M., M. D., formerly Professor of Electro-Therapeutics in the New York Post-Graduate Medical School and Hospital, etc., etc. With formulas. Fourth edition. Octavo, pp. xii.—294. Price, $2.75. New York: E. B. Treat, 5 Cooper Union. 1895.

The popular demand for this book is indicated by the fact that four editions have appeared in a little more than ten years. Its earlier editions have received commendatory notices at the hands of the Journal, hence there is no occasion for extended review at this time. The class of subjects that suffer from sexual neurasthenia deserves not only the greatest sympathy but the kindliest attention that science can bestow. Many a useful hint in the management of such sufferers can be obtained from a careful reading of this volume. In former editions there is nothing to cover a class of cases that is in a state of continual sexual excitement. Therefore, the editor has added a chapter to this edition entitled sexual erethism.

The book is uniform in size and style with the medical classics which Mr. Treat has been publishing for some years.

---

Twentieth Century Practice. An International Encyclopedia of Modern Medical Science. By leading authorities of Europe and America. Edited by Thomas L. Stedman, M. D., New York City. In twenty volumes. Volume II. Nutritive Disorders. New York: William Wood & Co. 1895.

The opening article of this volume treats of Addison's disease and other diseases of the adrenal bodies, and is written by Sir Dyce Duckworth, of London. It contains the latest information regarding these somewhat obscure diseases. The next section is devoted to the consideration of diabetes mellitus and is written by Professor Carl von Noorden, of Frankfort. The subject of treatment is here very ably handled and will be a welcome contribution to the literature of the subject.

The third division consists of an exhaustive monograph on rheumatism, from the pen of Dr. T. J. Maclagan, of London, who is the well-known originator of the salicylic treatment of this disease, hence his contribution will be eagerly sought. The fourth department is given up to the consideration of gout, to which over 200 pages is set apart. It is written by Dr. Henry M. Lyman, of Chicago, and is the most noted contribution to the literature of this disease that has yet appeared.

The fifth section treats of arthritis deformans and is written by Dr. Archibald E. Garrod, of London. Here is a subject, the literature of which is great while our knowledge of it is small. Dr. Garrod has done something, however, to increase it.

The next title is by Dr. Dujardin-Beaumetz, of Paris, lately deceased. The subject of it is diseases of the muscles, to which less attention has been given than the subject deserved, but this distinguished clinician has added much to our knowledge of it.

Finally, Professor M. J. Oertel, of Munich, presents an interesting dissertation on the subject of obesity, which is almost next in importance to the wasting of the human body. An excellent index serves to make the volume one of easy and ready reference. It is commended to the profession as a valuable collection of monographs, setting forth the latest information on the diseases which it considers.

---

A Manual of Bandaging. Adapted for Self-Instruction. By C. Henri Leonard, A. M., M. D., Professor of the Medical and Surgical Diseases of Women, and Clinical Gynecology in the Detroit College of Medicine. Sixth edition, with 139 engravings. Cloth, 8vo, 189 pages. Price, $1.50. Detroit, Mich.: The Illustrated Medical Journal Co., Publishers.

This book is so popular as to demand a sixth edition. Its chief claim to favor is in the brevity and clearness of the text, but especially in the excellence of its illustrations. To bandage well is an art, and every young physician should cultivate it. This book will prove to him a great aid in perfecting this important surgical accomplishment.

---

Antisepsis and Antiseptics. By Charles Milton Buchanan, M. D., Professor of Chemistry, Toxicology and Metallurgy, National University, Washington, D. C., with an introduction by Professor Augustus C. Bernays. Duodecimo, pp. 368. The Terhune Company: Newark, N. J. 1895.

This small volume presents in convenient form a condensation of the views of the best exponents of asepsis and antisepsis as understood at the present time. A practical knowledge of antiseptic technique is absolutely necessary to the successful practice of surgery, gynecology and obstetrics. Moreover, it is important that every physician shall be well versed in the knowledge of cleanliness as implied by the best surgical definition of the term. Infection must be prevented if possible. This book gives a history in its first four chapters of the subject from the earliest times down to the present. In other seven chapters it deals with the practical aspects of asepsis and antisepsis. It deserves to be well read, but it is a pity that it is so poorly printed. The least said about its engravings the better.

---

Suggestions to Hospital and Asylum Visitors. By John S. Billings, M. D., Director of the Hospital of the University of Pennsylvania, and Henry M. Hurd, M. D., Superintendent of the Johns Hopkins Hospital. With an introduction by S. Weir Mitchell, M. D. Price, 50 cents. Philadelphia: J. B. Lippincott Co. 1895.

This manual is based upon the idea that most hospital visitors are ignorant of their duties, at least when first appointed, which is undoubtedly correct. Most such will be glad to possess this little book, which will give them valuable hints, and aid in removing embarrassment when they begin to exercise their functions. It is written by experienced men, and is of a size convenient for the pocket.

---

The Physicians' Vade Mecum. Being a Hand-book of Medical and Surgical Reference, with other Useful Information and Tables. By Sebastian J. Wimmer, M. A , M. D., Author of Tables and Notes on Human Osteology, etc. With additions by Frank S. Parsons, M. D. Price, $1.00. Philadelphia: The Medical Publishing Co. 1894.

This little work is full of useful information, condensed and made available for ready reference. It contains many hints for carrying out simple procedures that are generally considered so common-place or so well understood as to need little or no elaboration in large treatises or text-books; hence, will find an easy place on the shelves of students, young physicians and druggists. The latter especially will find it of great benefit.

---

Dose-Book and Manual of Prescription-Writing, with a list of the official drugs and preparations and also many of the newer remedies now frequently used, with their doses. By E. Q. Thornton, M. D., Demonstrator of Therapeutics, Jefferson Medical College, Philadelphia. Saunders' New Aid Series. Small 8vo, pp. 334. Philadelphia: W. B. Saunders. 1895. Price, cloth, $1.25 net.

It would be very much to the credit of medical colleges if faculties would pay more attention to teaching proper methods of writing prescriptions. Physicians with fair classical culture often make sorry work of prescription writing, mixing subject and verb, singular and plural, nominative and accusative in inextricable confusion. The aid to be obtained from this book is valuable and deserves to be studied carefully by every one who may be in doubt with reference to the proper construction of prescriptions. Its tables of weights and measures, English and metric systems, dosage, official and officinal drugs and preparations and description of newer remedies afford excellent means of reference. All physicians find it necessary to refresh their memories upon the subjects herein contained.

---

### BOOKS RECEIVED.

A System of Surgery. By American Authors. Edited by Frederic S. Dennis, M. D., Professor of the Principles and Practice of Surgery, Bellevue Hospital Medical College, New York ; President of the American Surgical Association, etc., assisted by John S. Billings, M. D., LL. D., D. C. L., Deputy Surgeon-General, U. S. A. To be completed in four imperial octavo volumes, containing about 900 pages, each with index. Profusely illustrated with figures in colors and in black. Volume I., 870 pages, 422 engravings and two colored plates. Price, per volume, $6.00 in cloth ; $7.00 in leather ; $8.50 in half morocco, gilt back and top. Full circular free to any address on application to the publishers. Philadelphia : Lea Brothers & Co. 1895.

Weekly Abstract of Sanitary Reports issued by the Supervising Surgeon-General, H. M. S. Volume IX., Numbers 1 to 52. Washington : Government Printing Office. 1895.

Immunity, Protective Inoculations in Infectious Diseases, and Serum-Therapy. By George M. Sternberg, M. D., LL. D., Surgeon-General U. S. Army, Ex-President American Public Health Association, Honorary Member of the Epidemiological Society of London, of the Royal Academy of Medicine at Rome, of the Academy of Medicine at Rio Janeiro, of the Société d'Hygiene, etc., etc. One volume, 325 pages, octavo, bound in extra muslin, beveled edges, uniform with the other volumes of our *Medical Practitioners' Library*. Price, $2.50 ; in flexible morocco, $3.25. New York : William Wood & Co. 1895.

